# Supplementary material for: BCI-Walls: A robust methodology to predict if conscious EEG changes can be detected in the presence of artefacts
Source: PLoS One. 2023 Aug 24;18(8):e0290446. doi: 10.1371/journal.pone.0290446 (PMC10449140; doi:10.1371/journal.pone.0290446)
Supplement: Attachment — Submitted filename: loc.pdf [file pone.0290446.s001.pdf]

PONE-D-22-30139

Bernd Porr, Lucía Muñoz Bohollo

We thank both reviewers for the very constructive feedback.
The manuscript has undergone substantial revision. We have rewritten the abstract, explained the BCI-methodology in detail and expanded the results section by 3 more figures where we now guide the reader step by step through the calculations of the BCI-wall leading to a statistical analysis of the results.

Reviewer 1

`1. Reviewer: The abstract does not reflect the work of the authors. It must be rewritten again. It is hard to understand the scientific and technical parts to calculate SNR-wall and why it can be considered as hard and measurable criterion to determine if a BCI experiment can detect conscious EEG changes or not.`

We agree that the abstract wasn't well written. We have completely re-written it and worked in particular the essence of the BCI-walls methodology. We now write:

> Brain computer interfaces (BCI) depend on reliable realtime detection of conscious EEG changes for example to control a video game. However, scalp recordings are contaminated with non-stationary noise, such as facial muscle activity and eye movements. This interferes with the detection process making it potentially unreliable or even impossible. We have developed a new methodology which provides a hard and measurable criterion if conscious EEG changes can be detected in the presence of non-stationary noise by requiring the signal-to-noise ratio of a scalp recording to be greater than the SNR-wall which in turn is based on the highest and lowest noise variances of the recording. As an instructional example, we have recorded signals from the central electrode Cz during eight different activities causing non-stationary noise such as playing a video game or reading out loud. The results show that facial muscle activity and eye-movements have a strong impact on the detectability of EEG and that minimising both eye-movement artefacts and muscle noise is essential to be able to detect conscious EEG changes.

`2. Reviewer: Applying ICA might be another way to evaluate the results of the proposed techniques.`

That is an excellent suggestion to look into the ICA. Indeed, if one uses a montage with many electrodes one can use the ICA to calculate the SNR and SNR-wall. We write now in the discussion:

> The ICA could also be used to determine the SNR for the BCI-wall methodology. Instead, we have chosen the P300 methodology to determine the pure consciously controlled EEG power because ICA requires by definition more than one electrode and we recorded from just one. Future research could also calibrate the SNRs obtained from the ICA with those from the P300 responses. Be it P300 or ICA they are in stark contrast to determining the SNR by paralysing a participant temporarily [15,18] which would certainly yield the best results but no doubt would raise substantial ethical concerns.
>
> So far ICA has only been suggested to determine the SNR but it can also be used to determine the SNR-wall because the minimum $\sigma^2_{r,\min}$ and maximum noise variances $\sigma^2_{r,\max}$ are readily available in the independent component(s) of the ICA carrying the noise. Since the ICA is a well-established standard tool for EEG offline analysis calculating both the SNR and SNR-wall could just be a simple additional step which then allows the robust evaluation of an experiment.

`3. Reviewer: The authors didn't explain the impact of the eye movements (especially 0.1-3Hz) too on SNR-wall.`

We now show the impact of the eye movements on the SNR-wall. We have added a filter scenario ($0.1-3$ Hz) with this frequency range. In the sub-section "EEG pre-processing" we have added:

> 2. Low frequency bandpass filtering between a $0.1-3$ Hz 4th order Butterworth filter and 50 Hz powerline bandstop. This frequency range is in the EEG delta frequency range and is in particular suitable to investigate the effect of low frequency artefacts on the ability to detect EEG such as eye-blink, eye-movements and movement artefacts from the cables.

In the new section "Walk-through of the calculation of the SNR and SNR-wall of one representative subject for both jaw clench and reading" we then discuss what happens if one focuses on this frequency range for both the jaw clench experiment and reading. In fact the representative subject #20 we have chosen to illustrate the eye-blink artefacts also blinked during the jaw-clench task and we write for the "jaw clench" task:

> 0.1 − 3 Hz, Fig. 4D-F: This low frequency bandpass filter covers the frequency range of eye-blinks and saccades. While in the original trace Fig. 4A these have not been apparent, now they are easily identifiable in Fig. 4D. In particular during the first 10 seconds the subject blinked repeatedly which has been marked with the "*". These artefacts drive up the difference between chunks of small noise power and large noise power which in turn increase the SNR-wall.

For the "reading" experiment we write:

> The filtering of the electrode signal between 0.1 − 3 Hz emphasises eye movements and movement artefacts and shows numerous saccade like voltage changes which are characteristic of reading where the eyes need to scan the page. The large spike around 40 sec is a movement artefact. Together these artefacts cause a large difference between the smallest noise power and maximum noise power which in turn leads to a noise wall which is again much higher than the SNR (Fig. 5F).

`4. Reviewer: The authors must discus more about statistical evaluation techniques to validate their BCI-wall aspect in several EEG datasets.`

This is a very valid request. However, we are not aware of any datasets containing the "ground truth", namely the pure conscious EEG change without EMG which requires to paralyse a subject. However, on the other hand the Noise Walls methodology has been derived analytically and have been proven mathematically to be true. The mathematics has been validated over and over again in Digital Communications. This means that, indeed, the problems here are measurement problems: inaccuracies arise only from the measurement process of the different signal- and noise variances, in particular the estimation of the pure EEG signal which cannot be determined without causing substantial discomfort to the participant. Given there are no other datasets so far available, we suggest a way forward and take up the suggestion by the reviewer to use the ICA to cross validate against the P300 response in future research. We discuss this rationale in the discussion section and write:

> The ICA could also be used to determine the SNR for the BCI-wall methodology. Instead, we have chosen the P300 methodology to determine the pure consciously controlled EEG power because ICA requires by definition more than one electrode and we recorded from just one. Future research could also calibrate the SNRs obtained from the ICA with those from the P300 responses. Be it P300 or ICA they are in stark contrast to determining the SNR by paralysing a participant temporarily [15,18] which would certainly yield the best results but no doubt would raise ethical concerns.

Reviewer 2

`1. Reviewer: The results shown in Figure 3 are not clearly explained.`

Indeed, they were not and we have now expanded the results section substantially so that the statistical analysis becomes clear. We guide the reader now step by step to it: we 1st focus on one representative subject, then show that SNR/ SNR-wall pairs are random variables over all subjects and then we calculate the SNR/SNR-wall pairs for all subjects and do a statistical analysis involving a t-test. Figure 3 is now Figure 6 because we now lead the reader step by step towards the statistical analysis using intermediate figures. We refer the reviewer to the starting point of the new narrative where we write:

1. **Investigating the raw data:** The different datasets (jaw clench, lying eyes closed/open, word search, Sudoku, phone app, reading, colouring) used in this study exhibit different signal and noise characteristics in their time- and frequency domain. We will use subject #20 as a *representative example* to point out the differences between the datasets.
2. **Walk-through of the calculation of the SNR and SNR-wall of one subject for both jaw clench and reading:** As instructional examples we calculate step by step the SNRs and SNR-walls of *one* subject for *jaw-clench* and *reading* at different EEG frequency ranges such as: full range, only low frequencies ($0.1-3$ Hz), wide bandpass ($8-18$ Hz), narrow bandpass ($8-12$ Hz) and derivative.
3. **Statistical analysis with t-test:** Using the same approach as in step 2 but for *all* subjects enables us to do a statistical analysis with a t-test which determines if the SNR of a task is *significantly* larger than the SNR-wall and thus conscious EEG changes can significantly be detected.

This then leads to the statistical analysis after having lead the reader through the representative examples, jaw clench and reading, which leads us also the next point raised by the reviewer:

`2. Reviewer: Specifically, the tasks involving jaw clenching and reading, which require movement of the facial muscles, are not elaborated. The authors should provide more details on how these tasks were performed and how they affect the SNR of the EEG data.`

We have now done an extensive analysis of the facial muscle activity and looked deeply into jaw clenching and reading. Fig 4 and the corresponding section now extensively explores the data generated during jaw clench and Fig 5 explores the data during reading.

`3. Reviewer: The authors did not provide a clear picture of how the SNR-wall method supports their proposed methodology. They should provide more detailed analysis and explanation of the results to support the efficacy of their approach.`

Thank you very much for this feedback. We have now provided a detailed analysis of the BCI-wall method. First, we have expanded the theory section which then leads to a clear 3 step process how to calculate the BCI-wall:

> Having now derived the underlying analytics we can devise a step-by-step guide on how to determine if EEG changes $c[n]$ can be detected at all:
>
> 1. **Calculate the SNR-wall** by using the minimum noise variance $\sigma^2_{r,\text{min}}$ and the maximum noise variance $\sigma^2_{r,\text{max}}$ of the brain recording (Eq. 20).
> 2. **Calculate the SNR** as the ratio of pure consciously controlled EEG power and noise power (Eq. 10).
> 3. **Compare the SNR with the SNR-wall:**
>
> $$\text{conscious EEG changes are detectable} = \begin{cases} \text{yes} & SNR > SNR_{\text{wall}} \\ \text{no} & \text{otherwise} \end{cases}$$
>
> In the following sections we call the entire three-step process of determining if conscious EEG can be detected "BCI-Wall".

We then use the cartoons in Fig 2 and guide the reader through the calculations again using the 3 steps. We write now:

> As outlined above, the BCI-wall calculations require three steps: 1) SNR-wall calculation, 2) SNR calculation and 3) comparing SNR and SNR-wall to determine if conscious EEG detection is possible. We are now describing how this can be done practically and is also an instructional example for other datasets. The corresponding Python code is available on GitHub [13].

Step 1: SNR-Wall

In order to calculate the SNR-wall we need to calculate ρ (Eq. 14) which is the ratio between the maximum $\sigma^2_{r,\text{max}}$ and minimum $\sigma^2_{r,\text{min}}$ noise power. To find these two values we use a sliding window of length $\tau = 2$ sec and calculate the noise power for different sample positions:

$$\sigma_{\text{chunk}}[n] = T(d[n \dots n + \tau])$$

and obtain its maximum and minimum over the entire recording of N samples: (see Fig. 2):

$$\begin{aligned} \sigma^2_{r,\text{min}} &= \min_{n=0 \dots N-1} \sigma_{\text{chunk}}[n] \\ \sigma^2_{r,\text{max}} &= \max_{n=0 \dots N-1} \sigma_{\text{chunk}}[n] \end{aligned}$$

With Eqs. 14 and 20 one can then calculate the SNR-wall which is usually expressed in dB to make it comparable to the signal to noise ratio (which is introduced in the next section):

$$SNR_{\text{wall}} = 10 \log_{10} \left(\rho - \frac{1}{\rho} \right) \tag{1}$$

Step 2: SNR

The SNR is calculated as a ratio between signal power $T(c)$ and the nominal noise power σ^2_r:

$$SNR = 10 \log_{10} \left(\frac{T(c)}{\sigma^2_r} \right) = 10 \log_{10} \left(\frac{T(c)}{\rho \sigma^2_{r,\text{min}}} \right)$$

where we can re-use the SNR-wall parameters ρ and $\sigma^2_{r,\text{min}}$ from the previous section to calculate the nominal noise power σ^2_r. Measuring the pure conscious changes of EEG c and its power $T(c)$ is only indirectly possible as it is not ethical to paralyse subjects. The P300 evoked potential offers an individual estimate of the consciously generated signal power $T(c)$ calculated from the peak P300 voltage. This will be used for estimating the power of the signal for both a time domain task (i.e. P300) and frequency domain task (i.e. motor imagination):

- **Time-domain power of the signal:** The P300 peak can be used straight away for a time domain calculation of its power (Fig. 2A):

  $$T_t(c) = c^2_{\text{max}}$$

  where the $T_t(c)$ is the power of the pure EEG in the time domain.

- **Frequency-domain power of the signal:** BCI systems using the frequency domain change the power of a narrow frequency band (Fig. 2B), for example by motor imagination. Here, we assume that:

- The EEG power generated in a narrow frequency band is comparable to the power generated in the time domain $T_t(c)$. This is indicated between the panels Fig. 2A and B by noting that the peak powers are identical.
- However, because motor imagination *reduces* power consciously the actual conscious power $T_f(c)$ is only as strong as the *reduction* of power. Here, we assume a 40% reduction of power.

Given that only the reduction is the conscious power change c and anything else will be absorbed in the noise term r we can calculate the pure signal power (in contrast to the noise) as:

$$T_f(c) = T_t(c) - T_t(c) \cdot 40\%$$

After having calculated the power of the signal $T_f(c)$ we now need to calculate the power of the noise. Given that the small P300 evoked potentials c are buried in the EEG d, one can assume that the noise variance containing a consciously generated signal and one without are basically identical [14]: $\sigma_r^2 \approx \sigma_d^2$. Thus we take the variance of the EEG epoch $d[n]$ (Fig. 2D) as

$$\sigma_r^2 \approx T(d)$$

where the power of the EEG epoch $T(d)$ is calculated with Eq. 7 which is the average power or variance.

Step 3: Comparing SNR and SNR-Wall: determining if conscious control can be detected

If the SNR of the EEG (Eq. 10) is above the SNR-wall (Eq. 20) then detection of the conscious EEG change c is possible. Otherwise not.

We have then added a complete walk-through of the BCI-wall methodology by using subject 20 as an example of how to calculate the SNR and SNR-wall. Again using the 3 steps above. We have added two more figures (4 and 5) and also updated Fig 2 and its step by step guide how to calculate the BCI-wall.

`4. Reviewer: The authors should provide further validation of their results by applying a statistical test such as t-test to assess the significance of their findings.`

We have applied a t-test to all results and indicated significance in the statistical results (Fig 6) with a "". The results of the statistical analysis are also discussed extensively in the text.*

`5. Reviewer: The paper could benefit from a more detailed comparison with existing methods to demonstrate the advantages of the proposed methodology.`

This is a very good suggestion and we have added it to the discussion. We now write:

> An existing method of how to determine if EMG interference is detrimental to EEG detection is to use the amplitude of a bandpass filtered electrode signal and suspend data collection when it is above a certain threshold [16]. Another suggested method is to use a montage consisting of a large number of electrodes and then apply independent component analysis (ICA) to the electrode signals [3, 16, 17] which separates noise from EEG which then allows the calculation of the SNR. The ICA could also be used to determine the SNR for the BCI-wall methodology. Instead, we have chosen the P300 methodology to determine the pure consciously controlled EEG power because ICA requires by definition more than one electrode and we recorded from just one.
