# Supplementary material for: BCI-Walls: A robust methodology to predict if conscious EEG changes can be detected in the presence of artefacts
Source: PLoS One. 2023 Aug 24;18(8):e0290446. doi: 10.1371/journal.pone.0290446 (PMC10449140; doi:10.1371/journal.pone.0290446)
Supplement: Attachment — Submitted filename: loc2.pdf [file pone.0290446.s002.pdf]

PONE-D-22-30139

Bernd Porr, Lucía Muñoz Bohollo

We thank reviewer 2 for the constructive feedback.

Reviewer 2

`1. Reviewer: The paper does not provide a clear explanation for how the SNR-wall threshold was selected, which could be a limitation.`

`Finally, the paper would benefit from a more detailed discussion of the reasons behind the selection of the SNR-walls and the SNRs for the different experimental conditions and tasks, which could help to clarify the rationale behind the BCI-Walls methodology and improve the overall quality of the paper.`

There is no need to select SNR-walls and SNRs at all because their values directly result from two formulas: the SNR-wall is directly calculated from the ratio between the min and max noise variances of the electrode signal (Eq. 20). The SNR itself is calculated by using the ratio between the conscious EEG changes and noise (Eq. 10). That is why this approach is so powerful as it is based on two formulas which cannot be tweaked towards a perhaps favourable outcome.
We have added this to the 1st paragraph of the discussion:

> In this paper we have introduced an objective hard criterion which determines if it is possible to detect conscious EEG changes in a recording contaminated with non-stationary noise. Specifically, this methodology requires the SNR of an electrode signal to be higher than its SNR-wall so that conscious changes in EEG can be detected. The SNR-wall is calculated by taking the ratio between the minimum and maximum noise variances of the electrode signal (Eq. 20). The SNR is calculated using the standard definition of SNR which is here the ratio between the consciously generated EEG component against the nominal noise level (Eq. 10). Both equations result from sound analytical calculations and were then used to calculate all SNR-wall and SNR values for all experimental conditions and tasks. In other words, no manual selections of the SNR-walls, SNRs or thresholds are needed for the BCI-walls framework protecting it from subjective, possibly arbitrary choices.

2. Reviewer: To enhance the reliability of the results, it would be beneficial to separately analyse each task in the experimental setup rather than combining all tasks together.

This is an excellent suggestion. The statistical analysis was ordered according to the different filtering techniques but lumped the tasks together. This was done because reviewer one was very interested in the different filtering techniques. However, we very much agree that a section is needed analysing each task. We have now added a subsection to the analysis where we go through every experimental setup / task and then describe under which filtering condition one can detect conscious changes in the EEG. We now have two sub-sections: a) analysis according to pre-processing and b) the new analysis according to different tasks. We have added to the text:

Table 1: **Detection of conscious EEG changes significantly possible ordered by task.**

	**A** $0.1 >$ Hz	**B** $0.1 - 3$ Hz	**C** $8 - 18$ Hz	**D** 10 ± 2 Hz	**E** d/dt
lying/EO			*	*	*
lying/EC				*	*
phone app			*	*	*
Sudoku			*	*	*
Word Search			*	*	*
Colouring			*	*	
Reading				*	
Jaw clench				*	

In the previous section we have ordered the results according to the *pre-filtering* of the electrode signal. Of equal interest is which *task* allows the detection of conscious EEG changes and which renders it impossible to do so. For this purpose we have re-ordered the results of Fig. 6 and show them task by task in table 1. As in Fig. 6 the "*" in table 1 indicate that conscious EEG changes can be significantly detected. The labels A-E match the panel labelling in Fig. 6 and are the different pre-filtering methods.

Lying down, eyes open: Conscious detection of EEG is possible here for moderate wideband prefiltering ($8 - 18$ Hz), narrow alpha band filtering (10 ± 2 Hz) or using the derivative which acts as a highpass. Lying flat down and having the eyes open mainly creates eye blink artefacts which, without any filtering, cause a high ratio between the minimum and maximum noise variances which in turn result in a high SNR wall. EEG detection is possible if these eye-blink artefacts are removed by a highpass filter characteristic.

Lying down, eyes closed: With lying down and eyes closed detection of conscious EEG changes is still possible when focusing on the

narrow alpha band or using the derivative as a highpass filter. However wider bandpass filtering yields no significant results. As mentioned above closing the eyes in a lab is slightly uncomfortable which seems to result in a higher muscle tone. Comparing Fig. 6C between eyes open and closed only the SNR changes but not the SNR-wall which means that the actual ratio of min and max noise variance is not different between those two tasks but rather the constant muscle tone which is reflected in a higher SNR while having the eyes closed.

Phone app, Sudoku, Word search: As outlined further above these three tasks have only moderately elevated non-stationary muscle activity and eye-movements. The muscle activity mostly arises from being a bit more tense when solving the tasks but causes only low ratios between highest noise variance and lowest noise variance which in turn results in low SNR-walls. Even the simple derivative which favours muscle noise still yields significant results. Again, the low frequency filtering and wideband filtering make it impossible to detect conscious EEG changes because the low frequency eye movements cause high SNR-walls.

Colouring: This is now a task which involves strong arm muscle activity but also most likely compensatory muscle activity in the entire body such as neck muscles. This results in higher muscle noise levels compared to the previous tasks and now the filtering with the derivative no longer allows detection of conscious EEG changes. However, wideband prefiltering ($8-18$Hz) and narrow alpha band filtering (10 ± 2 Hz) still allow significant EEG detections.

Reading aloud: This task causes irregular contraction of facial muscles and the eyes need to scan the text which cause eye artefacts as well. This non-stationary noise leads to high SNR walls which prevent the detection of conscious EEG changes except when performing narrow band filtering around the alpha band. This filtering steers clear of both eyeblink artefacts and higher frequency muscle noise as much as possible.

Jaw clenching: The jaw muscles create very high amplitude bursts of muscle activity which are about 50 times higher than the baseline noise. Without any filtering this results in high ratios of minimum and maximum noise variances which in turn results in very high noise walls. Similarly to reading aloud removing the non-stationary muscle noise is crucial to be able to detect conscious EEG changes and is only possible if detection focuses just on the narrow alpha band.

Overall tasks with strong non-stationary facial muscle activity such as reading aloud or jaw clenching allow only significant detection of conscious EEG changes if one performs narrow band filtering around the alpha band which steers clear as much as possible of both muscle noise

and eye movement artefacts. However, eye movement artefacts are less of a problem than muscle artefacts because their frequencies do not overlap with the standard BCI frequency detection ranges. Those tasks which contain mostly eye movements but little muscle noise such as playing a video game allow detection of conscious EEG changes with a wideband BCI bandpass ($8 - 18$Hz) but also with a derivative which only attenuates eye-movements but not muscle noise.

3. **`Reviewer: While the paper proposes a validation method for the unknown dataset, the validation is only performed on a limited set of data with fixed amplitudes.`**

The dataset is as diverse as it can get ranging from very low amplitudes while lying down to very high amplitudes during jaw clenches. We'd like to draw the reviewer's attention to Fig 3. The amplitudes are very different between the different tasks. For example the difference between "Lying eyes closed" and "Jaw clench" is more than 50-fold. Note we had to use even different y-scales between "Jaw clench" and "Lying eyes closed". The jaw clench reaches nearly an amplitude of 1 mV while lying down has amplitudes of about 20 μV. In the paper we make this now clear in the introduction to Fig 3:

> We are now going through the different tasks step by step. We start with "jaw clenching" followed by "lying down, eyes closed" being the tasks with the largest difference in noise amplitudes where jaw clenching has about 50 times more noise than lying down. Note that, for the noise wall calculation, it is the *ratio* between the highest and lowest noise variances that are used (Eq. 25), not the absolute amplitudes. The ratio is again highest for the jaw clench:

In the section "Data acquisition" we explain why we have chosen the tasks such as jaw clench, lying down and reading – namely that we have a diverse dataset with different noise profiles. Note that the ratio between a high noise variance and low noise variance defines the noise wall value. We write there:

> For example lying down with eyes closed will have quite similar maximum and minimum noise variances resulting in a low SNR-wall. However, while reading out loud facial muscles will create short bursts of strong muscle noise as well as eye movement artefacts and the ratio between smallest and highest noise variance will be large which in turn will result in a high SNR-wall. Consequently, we have devised different tasks ranging from low noise variance ratios while lying down to extreme noise variance ratios during jaw clench.

4. Reviewer: It would be helpful to validate the methodology on a larger and more diverse dataset, including EEG signals with varying amplitudes. Statistical analysis results are also suggested to mention.

a) *As outlined above the dataset is as diverse as it can get and we designed the tasks to have as much difference in the EEG amplitudes as possible. Creating a substantially larger dataset is beyond the scope of this paper as this would require certainly a new research grant, ethics, subject recruitment of a large cohort and would delay this paper for years. In this paper we have recruited enough subjects to do meaningful statistics to be able to determine statistical significance. Thus, for this paper providing an instructional example a larger dataset is not required but hopefully it will inspire future research with larger number of subjects.* ***b)*** *Validation requires the knowledge of the ground truth: the true SNR and the true noise wall. We kindly ask the reviewer to read our replies to reviewer one of the previous revision round who suggested to cross validate our approach with ICA. Their suggestions have been incorporated into the manuscript. However, this is far beyond the scope of this paper as this requires new experiments to cross validate between P300 and ICA.*

5. Reviewer: Furthermore, Figure 3 is missing the x-axis and y-axis labels, which could make it difficult for readers to interpret the data being presented. Adding clear labels would enhance the readability and interpretation of the figure.

Fig 3 has actually labels but we agree that this can easily be overlooked but all axis are labelled but only at the top as they are identical for every column in a panel. Since all columns in panel A are $d[n]$ and in microvolt the labelling was done at the very top only once and then to save space not done for every row. However, we have added the labels to the caption to make it clear they are always the same:

> **Complete dataset of a representative recording (subject 20).** A) Time domain of the different recordings ($d[n]$ in μV against s). B) Power spectra of $d[n]$ of the corresponding recordings (V^2/Hz). C) Raw P300 recording (μV against s). D) Averaged P300 response (μV against ms).
